# Transcriptional and Proteomic Analysis of the *Aspergillus fumigatus ΔprtT* Protease-Deficient Mutant

**DOI:** 10.1371/journal.pone.0033604

**Published:** 2012-04-13

**Authors:** Shelly Hagag, Paula Kubitschek-Barreira, Gabriela W. P. Neves, David Amar, William Nierman, Itamar Shalit, Ron Shamir, Leila Lopes-Bezerra, Nir Osherov

**Affiliations:** 1 Department of Clinical Microbiology and Immunology, Sackler School of Medicine Ramat-Aviv, Tel-Aviv, Israel; 2 Department of Cellular Biology, The Roberto Alcantara Gomes Institute of Biology, University of Estado Rio de Janeiro, Brazil; 3 Department of Computer Science, Tel-Aviv University, Ramat-Aviv, Tel-Aviv, Israel; 4 The J. CraigVenter Institute, Rockville, Maryland, United States of America; 5 Sackler School of Medicine, Ramat Aviv, Tel-Aviv, Israel; Montana State University, United States of America

## Abstract

*Aspergillus fumigatus* is the most common opportunistic mold pathogen of humans, infecting immunocompromised patients. The fungus invades the lungs and other organs, causing severe damage. Penetration of the pulmonary epithelium is a key step in the infectious process. *A. fumigatus* produces extracellular proteases to degrade the host structural barriers. The *A. fumigatus* transcription factor PrtT controls the expression of multiple secreted proteases. PrtT shows similarity to the fungal Gal4-type Zn(2)-Cys(6) DNA-binding domain of several transcription factors. In this work, we further investigate the function of this transcription factor by performing a transcriptional and a proteomic analysis of the *ΔprtT* mutant. Unexpectedly, microarray analysis revealed that in addition to the expected decrease in protease expression, expression of genes involved in iron uptake and ergosterol synthesis was dramatically decreased in the *ΔprtT* mutant. A second finding of interest is that deletion of *prtT* resulted in the upregulation of four secondary metabolite clusters, including genes for the biosynthesis of toxic pseurotin A. Proteomic analysis identified reduced levels of three secreted proteases (ALP1 protease, TppA, AFUA_2G01250) and increased levels of three secreted polysaccharide-degrading enzymes in the *ΔprtT* mutant possibly in response to its inability to derive sufficient nourishment from protein breakdown. This report highlights the complexity of gene regulation by PrtT, and suggests a potential novel link between the regulation of protease secretion and the control of iron uptake, ergosterol biosynthesis and secondary metabolite production in *A. fumigatus*.

## Introduction

*Aspergillus fumigatus* is a saprophytic mold which grows naturally on degrading organic materials. Its small-sized conidia can easily reach the pulmonary alveoli by inhalation and cause a variety of pathological conditions [1]. Invasive Pulmonary Aspergillosis (IPA) is considered the most severe condition, threatening the lives of immunocompromised patients [2]. Infection can occur when the compromised immune system fails to eradicate the conidia from the lungs allowing germination, colonization and eventually penetration of the fungus through the pulmonary epithelium into the blood stream [3].

The ability of *A. fumigatus* to infect and persist inside the body has been attributed to an array of factors including: conidial pigmentation, secreted toxins, surface and cell-wall components, the ability to endure hypoxia, an efficient iron-uptake system and the secretion of proteases [4], [5]. In human A549 alveolar epithelial cells, culture filtrates (CFs) of *A. fumigatus* have been shown to disrupt the actin cytoskeleton, induce the production of proinflammatory cytokines and activate NFkB signaling. The addition of serine protease inhibitors to the secreted CF prevents these cellular events, suggesting that they are directly dependent on secreted fungal proteases [6], [7]. Recently, it has been shown that *A. fumigatus*-secreted proteases degrade complement proteins, which may serve as a mechanism for partially evading the immune defenses [8], [9].

Studying the process by which protease production is modulated led to the characterization of a proteolysis-deficient strain of *A. fumigatus* in which the transcription factor PrtT, a positive regulator of secreted proteases, is disrupted. The *ΔprtT* mutant exhibits a reduction in the transcription of secreted proteases and subsequently, reduced proteolytic activity of the CF. *ΔprtT* CF showed reduced killing of A549 lung alveolar cells and erythrocyte lysis [10], [11], [12]. However, the virulence of the *ΔprtT* strain was not attenuated in a murine model of IPA. The reconstituted *prtT* strain showed WT features under all examined conditions, validating that the phenotype of the *ΔprtT* mutant is the result of disruption of the *prtT* gene alone.

To better understand the role of PrtT in the control of gene expression and its effect on the secretome, we performed a combination microarray- and proteomics-based secretome analysis of the *ΔprtT* mutant. Microarray analysis has been used previously in *A. fumigatus* to identify the putative downstream targets of several transcription factors, including CrzA, SrbA, LaeA, SreA, HapX, AcuM, BrlA and StuA [13], [14], [15], [16], [17], [18], [19]. Proteomic analysis of *A. fumigatus* mutants in which key transcription factors are deleted has only been performed for *AfYap1*, involved in defense against reactive oxygen species [20].

Here we describe novel and unexpected findings showing that in addition to activating the expression of key secreted proteases, PrtT is also involved in regulating the transcription of genes involved in iron uptake, ergosterol biosynthesis and secondary metabolite biosynthesis. At the secretome level, deletion of PrtT not only reduces the secretion of key proteases but also alters the expression pattern of other apparently unrelated secreted proteins. Our work highlights the complexity of transcriptional regulation by PrtT.

## Materials and Methods

### Strains and culture conditions

*A. fumigatus* strain Af293, originally isolated at autopsy from a patient with IPA, and the *A. fumigatus prtT* disruption mutant (*ΔprtT*) derived from Af293, were used throughout this study [11]. Generation of the *ΔsidA* mutant strain was as described by Schrettl et al. [21]. For continuous growth, the different *A. fumigatus* strains were grown on YAG medium, which consists of 0.5% (wt/vol) yeast extract, 1% (wt/vol) glucose, and 10 mM MgCl_2_, supplemented with trace elements, vitamins, and 1.5% (wt/vol) agar when needed [22]. Skim milk (SM) medium consisted of 1% (wt/vol) glucose, 1% or 0.1% (wt/vol) SM (Difco, Livonia, MI), 0.1% (wt/vol) Casamino Acids (Difco), 7 mM KCl, 2 mM MgSO_4_ and 50 mM Na_2_HPO_4_-NaH_2_PO_4_ buffer (pH 5.3), supplemented with vitamins, trace elements (including 4 µM FeCl_2_), and 1.5% agar when needed [23]. Where indicated, ferrozine (Sigma Aldrich Corp., St Louis, MO) was added to the media 24 h prior to use. Conidia were harvested in PBS and counted with a hemocytometer.

### RNA extraction

Total RNA was isolated from each strain using the QIAGEN RNeasy Plant Kit (QIAGEN Inc. Valencia, CA) following the protocol for filamentous fungi. The RNA was digested with Turbo-DNase (Ambion, Austin, TX) according to the manufacturer's instructions.

### Sample preparation for microarray analysis

1×10^7^ conidia (WT or *ΔprtT* strains) were grown in 50 ml of 1% SM medium for 24 h at 37°C in an orbital incubator at 180 rpm. The mycelium was harvested using miracloth (Calbiochem, San- Diego, CA) and subsequently freeze-dried. Total RNA from three independent biological repeats was extracted, assessed for purity by the Agilent Bioanalyzer and shipped in isopropanol to the Nierman laboratory (JCVI) for microarray analysis.

### Microarray Analysis

Transcriptional profiling in this study was achieved using the *A. fumigatus* (Af293) DNA amplicon array containing 9516 genes as described previously [24]. The expression profiles were analyzed using EXPANDER, a general microarray analysis software [25]. EXPANDER supports all analysis steps, including normalization and filtering, gene clustering and differential expression analysis, and various statistical tests for gene group analysis including functional enrichment and transcription factor binding site enrichment. Differential genes were defined as genes whose expression level was altered by at least twofold, in at least two out of the three repeats. This criterion yielded a group of 229 upregulated genes and a group of 199 downregulated genes (Supporting Information S1). For each of these groups GO functional enrichment was evaluated using TANGO (FDR <0.05). Enrichment for other gene functional classes such as secreted proteases [26] , iron uptake [14], [27] and ergosterol biosynthesis [24] was performed using a hyper geometric test (Bonferroni corrected <0.05). Microarray datasets were deposited at Gene Accession Omnibus [28], accession numbers GSE33254).

### Quantitative real-time (qRT)-PCR

Conidia were grown in SM medium and total RNA was prepared as described above. When indicated, ferrozine (Sigma) or voriconazole (Pfizer, NY, USA) was added to the media at a final concentration of 1 mM and 0.125 µg/ml (half MIC), respectively.

RNA concentration was assessed using a NanoDrop^TM^ 1000 Spectrophotometer (Thermo Scientific Inc. Barrington IL) and 1 µg was taken for the RT reaction using StrataScript Reverse Transcriptase (Stratagene; Cedar Creek, TX). Gene-specific primers for expression analysis are listed in Supporting Information S2, Table A. Whenever possible the primers were located on the junction between exons or in different exons. As a standard control, reactions using primers specific for the *β*-tubulin gene (AFUA_1G10910) of *A. fumigatus* were performed. qRT-PCR was performed on an ABI Prism PCR- HT7900 using 500 nM primers and Power SYBR green PCR Master Mix (Life Technologies Inc. Carlsbad CA). All reactions were performed in triplicates, and the mixture included a negative no-template control.

**Figure 1:**
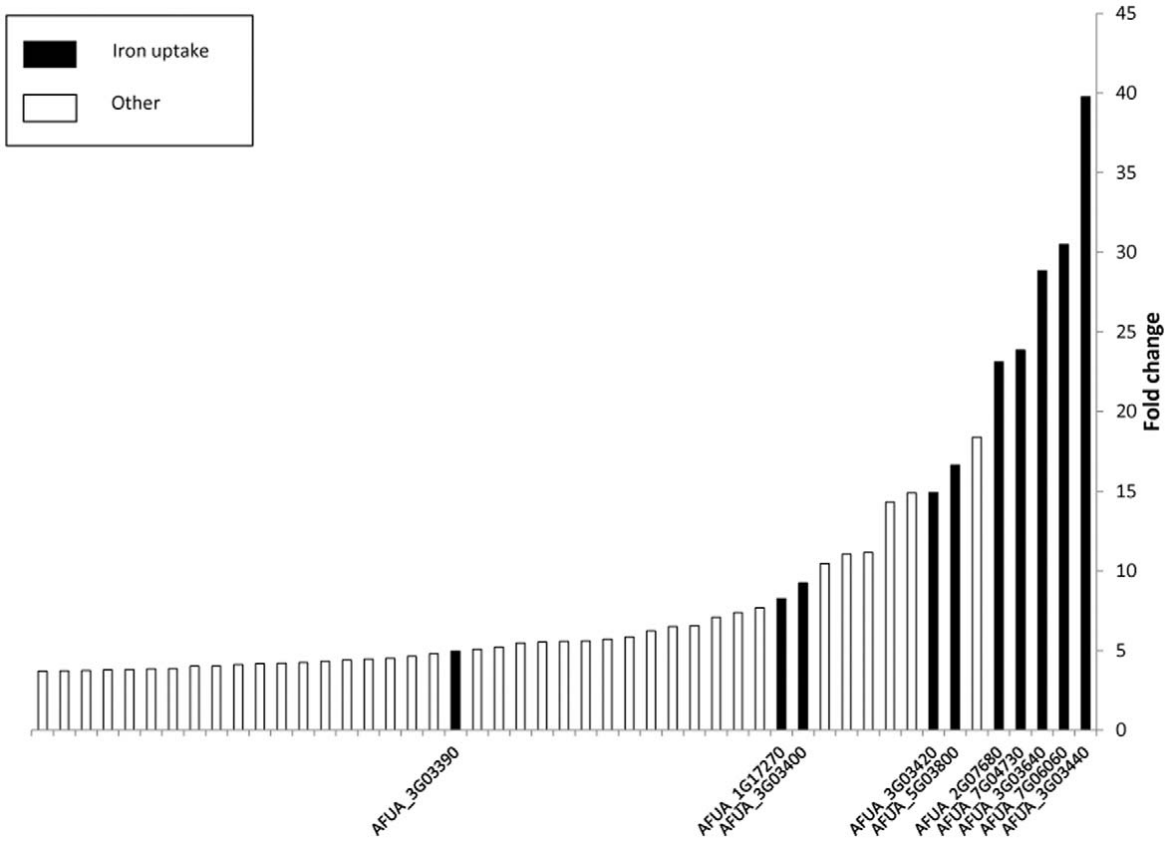
Top 50 downregulated genes plotted in ascending order by fold change. Black bars are the iron uptake genes, white bars are genes with various other annotations.

### Growth in iron-limited media

*A. fumigatus* wild-type (WT) and *ΔprtT* mutant strains were grown on 0.1% SM agar plates lacking iron and supplemented with different concentrations of the ferrous iron chelator, ferrozine. Conidia were point-inoculated on the agar using a toothpick dipped in PBS containing 1×10^6^ conidia/ml. Plates were incubated for 48 h at 37°C and the diameter of the colonies measured. In the positive control, the medium was supplemented with iron (8 µM FeCl_2_).

### Evaluation of siderophore production

A modification of the chrome azurol S (CAS) assay was used to detect siderophore production in *A. fumigatus* [29], [30]. This assay is based on competition for iron between the ferric complex of an indicator dye, CAS, and a chelator or siderophore. The iron is removed from CAS by the siderophore, which has a higher affinity for iron (III). This reaction results in a color change of the CAS reagent (usually from blue to orange). Conidia were grown at a concentration of 1×10^6^ conidia/ml in 1 ml of 1% SM medium in 24- well plates for 48 h at 37°C. The 1% SM medium contained iron (FeCl_2_ 8 µM) or no iron supplemented with the ferrous iron chelator ferrozine (1 mM). Culture supernatants were collected after 48 h and 100 µl was added to wells that were cut out of the CAS agar plates. Plates were incubated at 37°C for 4 h. The change in color or the presence of a halo around the well after incubation indicated siderophore production.

### Sensitivity to voriconazole by agar dilution

*A. fumigatus* WT or *ΔprtT* mutant strains were grown on 1% SM agar plates supplemented with different concentrations of voriconazole. Conidia were point-inoculated on the agar using a toothpick dipped in PBS containing 1×10^6^ conidia/ml. Plates were incubated for 48 h at 37°C and the diameter of the colonies measured.

**Table 1:** Selected enriched downregulated gene classes in the *ΔprtT* mutant vs. WT^1^.

Enriched class	Gene ID	Gene description	Fold change
**Secreted proteases P = 0.002**	AFUA_4G11800	alkaline serine protease Alp1	−14.3
	AFUA_6G00310	serine carboxypeptidase (CpdS),	−3.6
	AFUA_7G04930	alkaline serine protease (PR1),	−2.5
	AFUA_2G17330	serine peptidase, family S28,	−2.2
	AFUA_8G07080	Elastinolytic metalloproteinase Mep	−2.1
**Iron uptake ** ***P*** ** = 1E–15**	AFUA_3G03440	MFS family siderophore transporter, putative	−39.8
	AFUA_7G06060	siderochrome-iron transporter (Sit1), putative	−30.5
	AFUA_3G03640	siderochrome-iron transporter (MirB), putative	−28.8
	AFUA_7G04730	siderochrome-iron transporter, putative	−23.9
	AFUA_2G07680	L-ornithine N5-oxygenase SidA	−23.1
**Sterol and Fatty Acid Biosynthesis ** ***P*** ** = 0.001**	AFUA_2G00320	sterol delta 5,6-desaturase,	−6.5
	AFUA_1G03150	c-14 sterol reductase	−4.2
	AFUA_4G06890^2^	14-alpha sterol demethylase Cyp51A, ERG11	−4.2
	AFUA_5G07780^2^	Squalene monooxygenase ERG1	−3.7
	AFUA_6G05140^2^	sterol delta 5,6-desaturase, ERG3	−2.9
**Cellular ketone metabolic process** ***P*** ** = 0.001**	AFUA_6G07720^3^	homogentisate 1,2-dioxygenase (HmgA), putative	−4.2
	AFUA_2G04220	phosphoenolpyruvate carboxykinase (ATP) (AcuF)	−4.0
	AFUA_4G06620	NADP-dependent malic enzyme (MaeA)	−3.5
	AFUA_2G08280^3^	proline oxidase (PrnD)	−3.5
	AFUA_5G04250^3^	fumarylacetoacetate hydrolase (FahA)	−3.3
**Oxidoreductase activity ** ***P*** ** = 0.008**	AFUA_1G17180	pyridine nucleotide-disulphide oxidoreductase, putative	−7.7
	AFUA_5G03930	alcohol dehydrogenase, putative	−3.8
	AFUA_6G13790	monooxigenase	−3.6
	AFUA_7G02010	hypothetical protein	−3.6
	AFUA_1G07480	coproporphyrinogen III oxidase,	−3.3
	AFUA_4G08710	short chain dehydrogenase,	−3.2
**Cytochrome C oxidoreductase activity ** ***P*** ** = 0.008**	AFUA_3G06190	Cytochrome c oxidase subunit Via	−2.6
	AFUA_3G14440	cytochrome c oxidase family	−2.6
	AFUA_2G03010	cytochrome c subunit Vb, putative	−2.5
	AFUA_5G10560	cytochrome c oxidase subunit V	−2.2

1Top five genes with the highest fold change are shown in each category.

2 Ergosterol biosynthesis pathway.

3 Involved in amino acid catabolism.

### Preparation of fungal CF for proteomics

*A. fumigatus* WT or *ΔprtT* mutant strains were grown at a concentration of 1×10^6^ conidia/ml in flasks containing 250 ml SM medium. Fungal cultures were grown in an orbital incubator for 48 h at 37°C, 180 rpm. Samples were strained using miracloth and the CF collected. CFs were dialyzed at 4°C against DDW, using a Cellulose Membrane (Cellu-Sep T2/Nominal MWCO: 6,000 – 8,000) for 24 h and then freeze-dried.

### SDS-PAGE

The secretome extracts of the WT and *ΔprtT* strains were first precipitated by 20% trichloroacetic acid/acetone (vol/vol),suspended in rehydration buffer (30 mM Tris, 7 M urea, 2 M thiourea, 4% wt/vol CHAPS) and protein concentration was determined either by the Bradford method or the BCA Protein Assay Kit (Thermo Scientific; Rockford, IL). Then, 10 µg of each secretome extract was separated by 12% SDS-polyacrylamide gel electrophoresis in a miniPROTEAN system (Bio-Rad laboratories Inc., Hercules CA). The 1D gels were stained with colloidal Coomassie [31] and gel pieces were excised for further protein identification.

### DIGE 2D-gel electrophoresis

Protein abundance was compared between the WT and *ΔprtT* secretomes using four replicates for each experimental condition. Each sample was minimally labeled with CyDyes (Cy3 or Cy5) according to the manufacturer's instructions (GE Healthcare, Waukesha WI). An internal pool generated by equal amounts of all extracts was labeled with Cy2. The focusing was performed using IPG strips (Immobiline DryStrip 3–11 NL, 18 cm), with the addition of 1,2% DeStreak and 1% IPG buffer 3–11 (GE Healthcare). Immobilized pH-gradient strips were reduced (1.5% wt/vol dithioerythritol) and alkylated (2.5% wt/vol iodocetamide) in equilibration buffer (6 M urea, 50 mM Tris-HCl, pH 6.8, 30% glycerol, 2% SDS). Equilibrated strips were run on homogeneous 12% polyacrylamide gels using an Ettan DALTsix electrophoresis system with low-fluorescence glass plates (GE Healthcare). All gels, after image analysis (described below), were stained by colloidal Coomassie [31].

**Table 2:** Selected enriched upregulated gene classes in the *ΔprtT* mutant vs. W.

Enriched class	Gene ID	Gene description	Fold change
**Transporter activity, GO:0005215 ** ***P*** ** = 0.008**	AFUA_8G00540^2^	hybrid polyketide synthase/nonribosomal peptide synthase, pseurotin biosynthesis	43.1
	AFUA_8G00370^2^	polyketide synthase, putative	27.7
	AFUA_8G00940	MFS drug transporter, aflatoxin exporter	21.0
	AFUA_8G00800	amino acid transporter, putative	9.9
	AFUA_2G09860	purine-cytosine permease	8.1
	AFUA_6G11840	sodium:bile acid symporter involved in azole resistance	5.4
	AFUA_4G01230	amino acid transporter, putative	5.2
	AFUA_6G03060	MFS monosaccharide transporter	5.0
	AFUA_1G12240	MFS peptide transporter, putaitve	4.6
	AFUA_5G11020	ammonium transporter	4.3
**Oxidoreductase activity, GO:0016491 ** ***P*** ** = 0.04**	AFUA_8G00480^2^	phytanoyl-CoA dioxygenase family protein	41.9
	AFUA_8G00560^2^	cytochrome P450, similar to SP:P79084:O-methylsterigmatocystin oxidoreductase (*Aspergillus flavus*)	38.1
	AFUA_8G00440^2^	steroid monooxygenase, putative	15.8
	AFUA_6G11850	hypothetical protein	11.4
	AFUA_4G14780^2^	cyp5081A1 cytochrome P450 monooxygenase, putative	10.3
	AFUA_5G08900	D-arabinitol dehydrogenase ArbD, putative	9.5
	AFUA_4G14800^2^	sdr1 short chain dehydrogenase, putative	9.5
	AFUA_1G04150	tartrate dehydrogenase	8.9
	AFUA_3G12960^2^	cytochrome P450 monooxygenase (GliC ortholog), putative	8.6
	AFUA_8G00510^2^	O-methylsterigmatocystin oxidoreductase, putative	7.0

1 Top 10 genes with the highest fold change are shown in every category.

2 Gene located in a gene cluster.

### 2D-DIGE image analysis

Protein spots were visualized by a Typhoon Trio variable mode Imager (GE Healthcare) using a resolution of 100 µm, and the quantification of protein expression was carried out with the DeCyder 7.0 software package (GE Healthcare). The Cy2 channel from each gel was used for normalization of the spot intensities. Inter-gel matching and statistical analysis were performed using DeCyder BVA (Biological Variance Analysis) module, and each comparison was filtered to find the spots having (a) P-value ≤0.05 and (b) greater than 1.5-fold change in expression between the groups. The Extended Data Analysis (EDA module) was used to perform the Principal Component Analysis (PCA) to identify underlying sources of variation [30].

### In-gel digestion and protein identification

Spots of interest were manually excised from 2-DE gels. The gel pieces were destained, shrunk, vacuum-dried and the peptides digested according to [32]. After digestion the samples were spotted on a MALDI target plate (Applied Biosystems) and mixed subsequently with matrix (α-cyano-4-hydroxy-trans-cinnamic acid, Sigma). The samples were analyzed with a 5800 Proteomics Analyzer MALDI-TOF/TOF mass spectrometer (Applied Biosystems, Foster City, CA) in manual mode. All mass spectra were externally calibrated with the 4700 Proteomics Analyzer Mass Standards Kit (Applied Biosystems). Peak lists from all MS and MS/MS spectra were submitted to database search using Mascot software (www.matrixscience.com). The samples were searched against the NCBInr database, against all taxonomies. Initial search parameters included two variable modifications: Carbamidomethyl (C) and Oxidation (M). Up to one missed cleavage site was allowed, peptide mass tolerance was 0.05 Da, and MS/MS tolerance was 0.2 Da. The access number and the name of the ORF were taken from the Universal Protein Resource server using the database UniProt Knowledge/Swiss-Prot. The prediction of a signal peptide (SignalP) in the sequence of identified proteins was investigated by the Fungal Secretome Knowledge Base (FunSecKB).

## Results

### Microarray analysis

We have previously shown that deletion of the gene encoding the *A. fumigatus* transcription factor PrtT, results in decreased transcription of secreted proteases and loss of secreted protease activity [11]. To better understand this process at the transcriptomic level, we determined changes in gene expression between *A. fumigatus* WT and *ΔprtT* strains by microarray analysis. WT or *ΔprtT* conidia were grown in SM for 24 h, harvested and RNA was extracted. SM medium was used because it induces strong protease secretion in the WT and none in the mutant [11]. There was no difference between the dry weight of the WT and *ΔprtT* strains at harvesting, suggesting that their growth rates after 24 h were similar. The 24 h time point was selected because we previously demonstrated that *prtT* mRNA levels are highest after 24 h of growth [11].

### Expression of genes involved in iron uptake and ergosterol biosynthesis is significantly reduced in the *ΔprtT* mutant

There was at least twofold decrease in the mRNA levels of 199 genes in the *ΔprtT* mutant strain relative to the WT (see Supporting Information S1). These genes were categorized by the Expander program and included, as expected, genes encoding secreted proteases (*p* = 0.002), but also, surprisingly, genes involved in iron uptake (*p* = 1E–15) and in steroid and fatty acid biosynthesis (*p* = 0.001). The *ΔprtT* mutant also showed significantly reduced transcript levels of genes involved in cellular ketone metabolic processes (*p* = 0.001) and transcripts encoding proteins with oxidoreductase activity (*p* = 0.008) in particular cytochrome C oxidoreductase activity (*p* = 0.008) (See Table 1 and Table B in Supporting Information S2, all reported terms are after FDR correction of 0.05). Remarkably, among the downregulated genes, those involved in iron uptake showed the most dramatic decrease (p = 1.64E–5 using Wilcoxon rank sum test). Fig. 1 depicts the top 50 downregulated genes in ascending order. This group contains 10 genes related to iron uptake that are strongly downregulated in the *ΔprtT* mutant (average fold change -20, in comparison to an average of −6.2 among the 40 other genes). The results suggest that in addition to activating the transcription of secreted proteases, PrtT may also be involved in the activation of iron-uptake genes, and in particular 8 of the 10 genes involved in non- reductive iron uptake by siderophores (*sidA*–*G*/siderophore biosynthesis, *Sit1/MirB/MirC*-siderophore transporters) and 2 out of the 3 genes involved in reductive iron uptake (*Fre2*-secreted ferric reductase, *FtrA*-permease) [14], [27]

**Table 3:** Significantly enriched physical clusters

Group	Cluster	*P*-value	#genes	Putative function
**Down regulated**	9	<E–7	9	Siderophore biosynthetic cluster
**Upregulated**	10	<E–6	7	ETP unknown Toxin Biosynthesis Cluster
**Upregulated**	15	<E–5	10	Unknown
**Upregulated**	22	<E–3	4	Unknown
**Upregulated**	24	<E–15	23	Pseurotin A biosynthetic cluster

**Figure 2:**
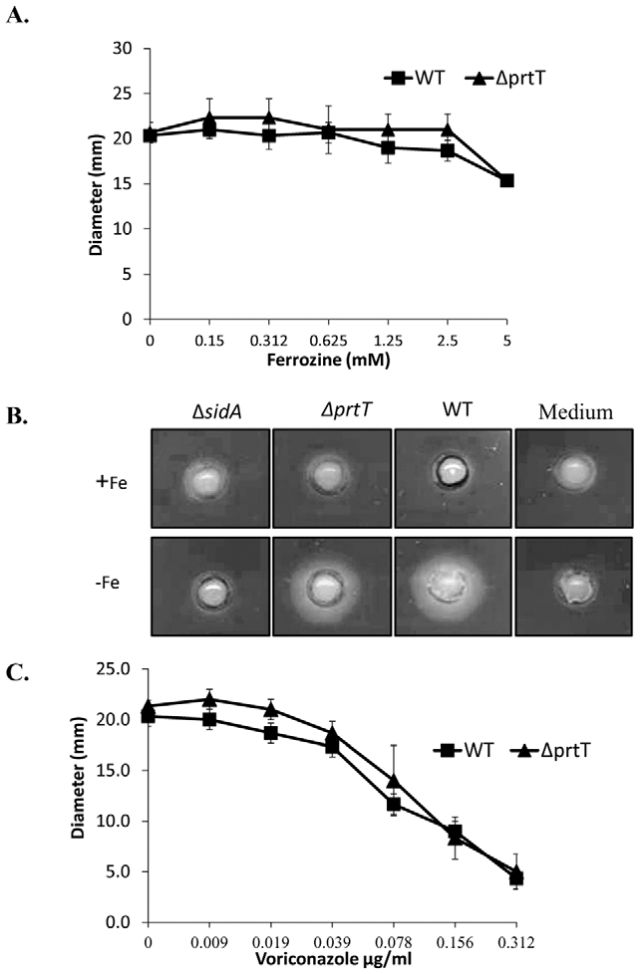
Phenotypic analysis of the *ΔprtT* mutant. (A) Growth in iron-limited media: WT and *ΔprtT* mutant were grown in iron-lacking 0.1% SM agar and supplemented with different concentrations of the ferrous iron chelator ferrozine. Colony diameter was measured after 48 h at 37°C. Similar results were seen with 1% SM and using liquid medium as well (data not shown). (B) Evaluation of siderophore production using the CAS assay. WT and *ΔprtT* conidia were grown either in liquid medium containing iron (indicated by +Fe) or on medium without iron (indicated by −Fe). Culture supernatants were collected and added to wells made in the CAS agar plates. The presence of a halo around the well indicates siderophore production. Non-inoculated growth media were also applied to wells on the CAS plate as a negative control (Medium) (C). Sensitivity to voriconazole. WT and *ΔprtT* mutant were grown on 1% SM agar plates supplemented with different concentrations of voriconazole. Colony diameter was measured after 48 h at 37°C.

**Figure 3:**
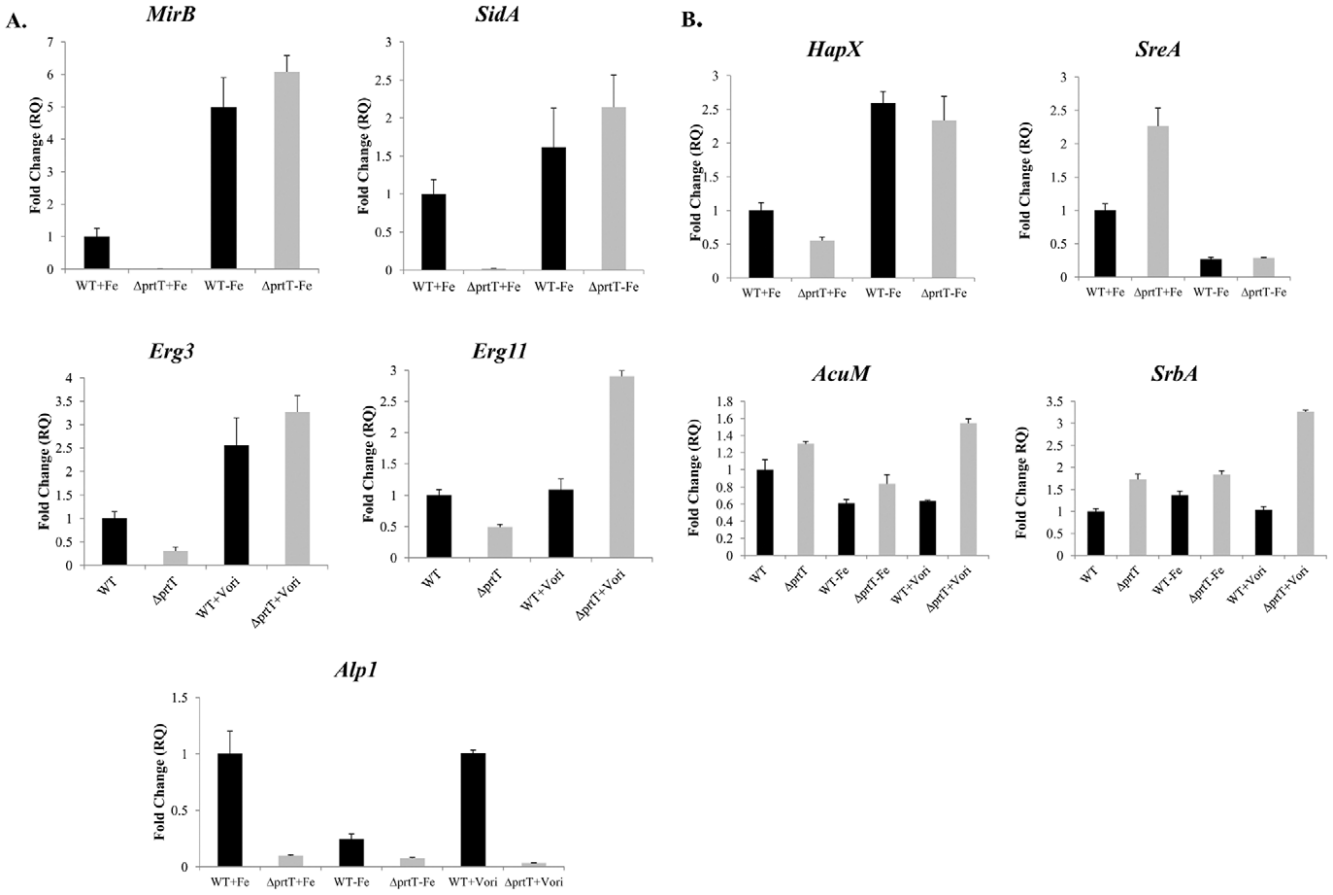
qRT-PCR evaluation of transcripts levels from genes that showed a differential expression in the microarray (A) representative genes (*MirB, SidA, Erg3, Erg11* and *Alp1*) and (B) the genes *HapX*, *SreA, AcuM* and *SrbA* encoding transcription factors that regulate iron uptake. Expression rates were normalized relative to mRNA levels of the β-tubulin-encoding gene (AFUA_1G10910) and set arbitrarily to 1 for the WT strain grown in 1% SM medium. Values are given in relative quantity of template compared to the original sample (RQ). RQ values were calculated by use of the equation: RQ  =  2^–ΔΔCT^, with ΔΔCT ± SD and ΔCT ± SDs. −Fe  =  growth medium lacking iron and containing 1 mM ferrozine. +Vori  =  growth medium supplemented with a sub-inhibitory concentration (0.125 µg/ml) of voriconazole. The experiment was repeated three times with similar results. Graphs show a representative experiment.

**Figure 4:**
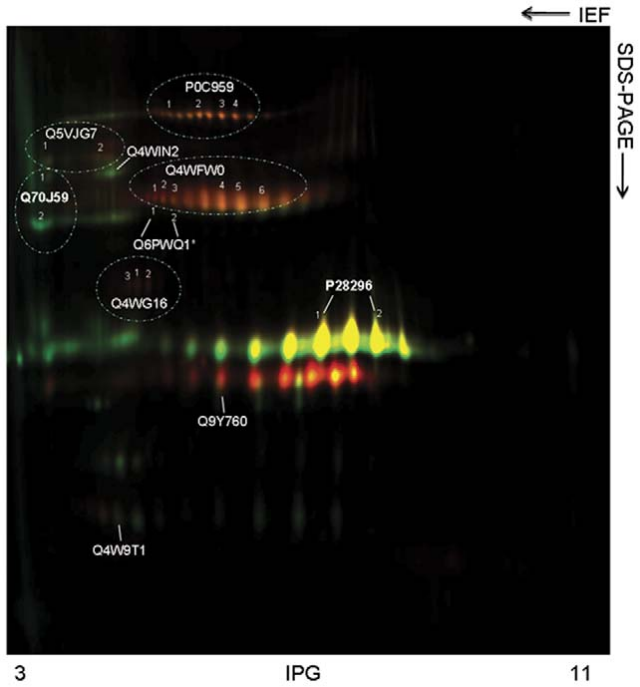
2-D DIGE comparing the secretomes of WT and *ΔprtT* strains. DIGE image overlay of WT (Cy3 label in green) and *ΔprtT* (Cy5 label in red) secretomes. Yellow spots indicate proteins detected in both strains and identified proteins are indicated by their accession number (SwissProt/UniProtKB database). Detected protein isoforms are represented with Arabic numerals.

Interestingly, 6 of the 11 genes categorized as participating in steroid and fatty acid biosynthesis and downregulated in the *ΔprtT* mutant are directly involved in the biosynthesis of ergosterol (hyper-geometric p-value = 1.8E–6) including the key pathway genes ERG1, ERG3, ERG11/Cyp51A,B (Table B in Supporting Information S2, footnote 1). Also notable was the finding that 8 of the 14 genes categorized as participating in cellular ketone metabolic processes and downregulated in the *ΔprtT* mutant are directly involved in amino acid breakdown, possibly due to a shortage of available amino acids upon reduced protease secretion by the mutant (Table 1 and Table B in Supporting Information S2, footnote 2).

### Expression of gene clusters involved in secondary metabolite biosynthesis is significantly increased in the *ΔprtT* mutant

There was a twofold or higher increase in the mRNA levels of 229 genes in the *ΔprtT* mutant strain relative to the WT (see in Supporting Information S1). These genes were categorized by the Expander program and included genes encoding transporters (*p* = 0.008) and proteins with oxidoreductase activity (*p* = 0.004) (Table 2 and Table C in Supporting Information S2). Surprisingly, a large number of the upregulated genes in the *ΔprtT* mutant are involved in secondary metabolite biosynthesis and transport (*p* = 1E–15, selected genes shown in Table 2 denoted by superscript^2^ and Table C in Supporting Information S2, denoted by an asterisk) and some are members of gene clusters previously described in Nierman et al. [24]. We therefore identified all of the genes in our microarray dataset that were significantly up- or downregulated in the *ΔprtT* mutant and located in a gene cluster (Table 3 and Table D in Supporting Information S2, all accepted terms are after FDR correction of 0.05). The results showed that nine genes of siderophore biosynthetic cluster 9 were downregulated in the *ΔprtT* mutant relative to the WT, whereas four clusters (10, 15, 22 and 24) were upregulated. The function of clusters 10, 15 and 22 is unknown. The genes upregulated in cluster 24 are involved in pseurotin A biosynthesis (Table 3 and Table D in Supporting Information S2). These results suggest that the *ΔprtT* mutant may produce more secondary metabolites than the WT when grown in SM medium.

### The *ΔprtT* mutant does not exhibit increased sensitivity to iron deprivation or to inhibition of ergosterol biosynthesis

The results of the microarray analysis, showing reduced expression of genes involved in iron uptake in the *ΔprtT* strain, suggested that it may be more sensitive to iron deprivation. We therefore compared the radial growth of the WT and *ΔprtT* strains in the presence of increasing concentrations of the ferrous iron chelator ferrozine (Fig. 2A). The *ΔprtT* strain was not more sensitive than the WT under iron limitation. Furthermore, the *ΔprtT* strain was not attenuated in its ability to secrete siderophores under iron limitation as measured by the CAS halo assay (Fig. 2B, compare halo diameters –Fe of WT and *ΔprtT* strain).

The results of the microarray analysis, showing reduced expression of genes involved in ergosterol biosynthesis in the *ΔprtT* strain, suggested that it may be more sensitive to azole antifungals which inhibit ergosterol biosynthesis. To test this possibility, we compared the radial growth of the WT and *ΔprtT* strains in the presence of increasing concentrations of voriconazole (Fig. 2C): again, the *ΔprtT* strain was not more sensitive than the WT in the presence of increasing concentrations of voriconazole.

**Table 4:** Secretome proteins identified by 2D−DIGE.

Identified Protein by *MS/MS*	ORF	Iso-form	Accesion number	MW theoretical (kDa)/pI	Signal Peptide	Expression rate- fold (ΔprtT/WT)
Secreted dipeptidyl peptidase Dpp V	AFUA_2G09030^1^	1,2,3,4	P0C959	79.6/5.59	Y	---
Pheromone processing carboxypeptidase (Sxa2)	AFUA_2G03510	1,2	Q5VJG7	59.7/4.77	Y	---
Tripeptidyl-peptidase (TppA)	AFUA_4G03490	1	Q70J59	65.7/5.3	Y	---
Tripeptidyl-peptidase (TppA)	AFUA_4G03490	2	Q70J59	65.7/5.30	Y	−5.23
Serine peptidase	AFUA_2G01250	--	Q4WIN2	58.5/4.86	Y	−1.95
FAD-dependent oxygenase	AFUA_3G00840^1^	1	Q4WFW0	54.9/6.52	Y	3.58
FAD-dependent oxygenase	AFUA_3G00840^1^	2	Q4WFW0	54.9/6.52	Y	2.67
FAD-dependent oxygenase	AFUA_3G00840^1^	3	Q4WFW0	54.9/6.52	Y	3.09
FAD-dependent oxygenase	AFUA_3G00840^1^	4	Q4WFW0	54.9/6.52	Y	2.02
FAD-dependent oxygenase	AFUA_3G00840^1^	5	Q4WFW0	54.9/6.52	Y	1.82
FAD-dependent oxygenase	AFUA_3G00840^1^	6	Q4WFW0	54.9/6.52	Y	1.89
Mannosidase I	AFUA_1G14560	1	Q6PWQ1	55.4/5.14	Y	3.58
Mannosidase I	AFUA_1G14560	2	Q6PWQ1	55.4/5.14	Y	3.09
β -1,3- glucanosyltransferase Bgt1	AFUA_1G11460	1	Q4WSV9	33/5.02	Y	2.96
GPI-anchored cell wall β-1,3-endoglucanase EglC	AFUA_3G00270^1^	1	Q4WG16	44.6/4.90	Y	2.96
GPI-anchored cell wall β-1,3-endoglucanase EglC	AFUA_3G00270^1^	2	Q4WG16	44.6/4.90	Y	5.13
GPI-anchored cell wall β-1,3-endoglucanase EglC	AFUA_3G00270^1^	3	Q4WG16	44.6/4.9	Y	2.75
Alkaline serine protease Alp1	AFUA_4G11800^1^	1	P28296	42.1/6.32	Y	−3.48
Alkaline serine protease Alp1	AFUA_4G11800^1^	2	P28296	42.1/6.32	Y	−3.46
Chitosanase	AFUA_4G01290^1^	--	Q9Y760	21.5/5.76	---	2.34
Conserved hypothetical protein	AFUA_4G03830	--	Q4W9T1	15.9/5.71	Y	4.35

1 genes also identified by Wartenberg ,et al. (43)

### Compensatory gene activation occurs in the *ΔprtT* mutant under stress

We hypothesized that the *ΔprtT* strain fails to show a sensitive phenotype under iron limitation or in the presence of voriconazole because of compensatory transcriptional activation of the genes involved in these pathways. We therefore used qPCR to analyze the mRNA levels of (i) *MirB* (siderophore transporter), *SidA* (siderophore synthesis) in response to iron limitation and (ii) *Erg11* (sterol demethylase), *Erg3* (sterol 5,6 desaturase) in response to a sub-inhibitory concentration (0.125 µg/ml ) of voriconazole (Fig. 3A). The level of *Alp1* protease mRNA, which we have previously shown to be strongly down-regulated in the *ΔprtT* strain [11], was used as a control Results showed that *MirB, SidA, Erg11, Erg3* and *Alp1* mRNA levels are strongly reduced in the *ΔprtT* strain in comparison to the WT when grown in SM liquid medium, independently validating the microarray results. However, in both WT and *ΔprtT* strains, *MirB/ SidA* and *Erg11/Erg3* mRNA levels were strongly increased in SM medium lacking iron, or containing voriconazole, respectively (Fig. 3A). In contrast *Alp1* mRNA levels were not increased in the *ΔprtT* strain under these conditions (Fig. 3A). Together, these results indicate that compensatory transcriptional mechanisms activated in the *ΔprtT* strain under iron limitation or inhibition of ergosterol biosynthesis are responsible for the lack of increased sensitivity to these treatments compared to the WT strain. Two transcription factors, HapX (activator) and SreA (repressor) are primarily involved in regulating iron uptake [14], [16]. We hypothesized that growth of the *ΔprtT* strain under iron limitation activates HapX and inhibits SreA expression, bypassing the need for PrtT. We therefore used q-PCR to analyze the mRNA levels of HapX and SreA in the WT and *ΔprtT* strain under normal and limiting iron levels. In SM medium containing normal iron levels, the *ΔprtT* strain expressed reduced levels of HapX activator and increased levels of SreA repressor compared to WT (Fig. 3B). This result (decreased activator, increased repressor) might explain the observed reduced expression of iron uptake genes in this mutant. In contrast, under iron starvation, the *ΔprtT* strain underwent a corrective compensatory response. It expressed elevated levels of HapX and reduced levels of SreA in a manner similar to the WT (Fig. 3B). This supports our hypothesis that under iron limitation the *ΔprtT* strain activates HapX and inhibits SreA expression, bypassing the need for PrtT and enabling it to grow like the WT under these conditions. Recently, two additional transcription factors, AcuM and SrbA were shown to activate transcription of HapX, increasing iron uptake and ergosterol biosynthesis [17], [33]. However, AcuM and SrbA mRNA levels were not altered in the *ΔprtT* strain compared to the WT, nor were they induced under limiting iron levels or the presence of sub-inhibitory concentrations of voriconazole (Fig. 3B). This result suggests that PrtT functions independently of AcuM and SrbA.

### Secretome analysis by 2D-DIGE

The proteins present in the secretome of the WT and *ΔprtT* strainswere evaluated by a 2D-DIGE quantitative proteomic analysis, followed by MALDI-TOF/ MS analysis.

The DIGE approach was used to quantify changes in protein expression of the most abundant species in both strains, because this technique shows high sensitivity for in-gel analysis of the differentially expressed proteins. This type of approach was applied because the aim was not to identify all proteins secreted but only the major differences in protein expression between the strains. The resultant gel shown in Figure 4 is representative of all five independent gels and five biologicql replicates which were used in the DeCyder analysis and the EDA mode analysis. All independent gels were imaged by scanning with different excitation wavelengths, producing protein profiles for each sample which were then overlaid, to enable exact matching of protein spots for the WT and *ΔprtT* secretomes (Fig. 4). Further analysis of gels using DeCyder software allows differentially expressed proteins to be accurately quantified. The 2D-DIGE analysis showed an average of 480 spots detected automatically by DeCyder software (Fig. 4). Among these, 94 spots were statistically validated using a differential abundance ratio of ≥ 1.5-fold and p<0.05. By this analysis, it was observed that the mutant strain overexpresses 63.8% of the proteins when compared with the WT. Only proteins present in at least 3 independent gels out of five, were considered to be differentially expressed. For protein identification the gels were further stained by colloidal *Coomassie* and the proteins identified as summarized in Table 4. Identified proteins are denoted by accession numbers and the protein isoform(s) are indicated by numbers (Fig. 4). All MS/MS data are presented in Table E in Supporting Information S2. In addition, the presence of signal peptides for all identified proteins was evaluated by FunSecKB (Fungal Secretome Knowledge Base). The *ΔprtT* mutant expressed reduced levels of ALP1 protease, TppA tripeptidyl peptidase and AFUA_2G01250 serine peptidase and increased levels of AFU_3G00840/FAD-oxygenase, AspF chitosinase, EglC endoglucanase and Bgt1 glucanosyltransferase (Table 4 and Fig. 4).

## Discussion

Deletion of the *A. fumigatus PrtT* gene encoding a C6-zinc finger transcription factor results in decreased transcription of genes encoding six secreted proteases and subsequent loss of secreted protease activity [11], [12]. To better understand the global role of PrtT in the control of gene expression and its effect on the secretome, we performed a combination of microarray and proteomics-based secretome analyses of the *ΔprtT* mutant.

The microarray analysis revealed several unexpected findings. First, expression of genes involved in iron uptake was dramatically decreased in the *ΔprtT* mutant under iron-replete (SM medium) conditions, suggesting that PrtT activates their transcription. It is unlikely that this is an indirect effect of nutrient starvation due to decreased protease secretion: SM contains sufficient glucose and amino acids and the growth rate of the *ΔprtT* mutant is similar to the WT in this medium. It is more likely that PrtT, in addition to activating transcription of secreted proteases, also upregulates iron uptake. Iron is needed for the activity of metalloproteases and oxidoreductases participating in the utilization of amino acids derived from protein hydrolysis. Therefore, under iron-replete protein-rich conditions, PrtT positively regulates genes involved in reductive iron assimilation and siderophore-mediated iron uptake, enabling efficient utilization of proteins as an energy source. Deletion of PrtT results in the down-regulation of these genes without leading to an observable iron-dependent phenotype probably because there is enough iron available in the SM medium for low-affinity uptake to suffice. We would, however, expect the *ΔprtT* mutant to exhibit reduced growth under iron-depleted conditions, but as we show in this report, this is not the case. Under these conditions, transcription of genes involved in siderophore-mediated iron uptake is strongly activated, probably as a result of compensatory activation of alternative transcription factors involved in iron uptake. Indeed we show that in the *ΔprtT* mutant under iron starvation HapX transcript levels increase while SreA levels decrease. This would result in both direct activation (via HapX) and derepression (via reduced SreA) of genes involved in iron uptake. Iron limitation therefore appears to activate a complex hierarchy of control elements, obviating the need for PrtT activity and ensuring that the organism reacts appropriately to the environmental challenge. Our findings suggest that PrtT operates as part of a larger network of transcription factors that is wired for functional redundancy.

At present, it is not known whether PrtT directly binds to the promoters of iron-uptake genes or whether it activates their transcription by an indirect mechanism. Our in-silico promoter motif analysis failed to identify significant conserved motifs in the promoters of the down-regulated genes (our unpublished data).

A second finding of interest is that deletion of *prtT* resulted in the upregulation of four secondary metabolite clusters (10, 15, 22 and 24), suggesting that PrtT negatively regulates their activity. Whereas the functions of clusters 10, 15 and 22 are unknown, cluster 24 contains genes for the biosynthesis of fumitremorgin (AFUA_8G00170- AFUA_8G00250) and pseurotin A (AFUA_8G00530- AFUA_8G00720) [13], [15], [34]. Only the genes involved in pseurotin A biosynthesis were upregulated in cluster 24 in the *ΔprtT* mutant. Pseurotin A is a competitive inhibitor of chitin synthase and is a neuritogenic agent [35]. Its expression is elevated under stressful conditions including hypoxia, during murine lung infection and following deletion of the global secondary metabolite regulator LaeA and the developmental transcription factor BrlA [13], [15], [36], [37]. Activation of secondary metabolite clusters in the *ΔprtT* mutant could be an indirect response to stress due to its inability to utilize proteins in the SM medium, or possibly a direct response resulting from interactions between PrtT and regulatory elements within the clusters. These possibilities remain to be examined.

The proteomic analysis identified 11 proteins secreted by the *A. fumigatus* WT strain when grown on SM medium. Of these, the *ΔprtT* mutant expressed reduced levels of ALP1 protease, TppA tripeptidyl peptidase and AFUA_2G01250 serine peptidase and increased levels of AFU_3G00840/FAD-oxygenase, AspF chitosinase, EglC endoglucanase and Bgt1 glucanosyltransferase compared to the WT strain. The increased expression of secreted polysaccharide-degrading enzymes in the *ΔprtT* mutant may indicates that it is (i) undergoing more intensive cell-wall remodeling than the WT or (ii) seeking an alternative carbon source because of its inability to utilize the proteins in the SM medium as an energy source.

Several groups have analyzed the secretome of WT *A. fumigatus* grown in minimal medium [38] or in the presence of elastin, collagen, keratin [39], [40] or fetal calf serum [41] as the main carbon/nitrogen source. Although there is significant variability between the secreted proteins identified in these studies, a core group of three secreted proteases; Alp1, Mep and DppV were induced in all. The most abundant secreted proteases were Alp1 and DppV which were also identified in this study. The other three proteases identified in our study (TppA, Sxa2 and AFUA_2G01250 serine peptidase) were not identified in the other studies. Of the six non-protease secreted proteins we identified, only three (Chitosanase/AFU_4G01290, EglC endoglucanase and FAD-dependent oxygenase/AFU_3G00840) were also identified by Wartenberg et al. [39].The large variations between the different studies may be due to differences in the proteomic analysis methodology, strain backgrounds, medium composition or length of culture.

Notably, a comparison between our transcriptome and proteome datasets revealed very little correlation between them. Only ALP1 protease and AspF chitosinase were significantly downregulated or upregulated, respectively, in both. Similar disparities have been shown for the yeast transcriptome and proteome datasets (Pearson correlation coefficient <0.4), and have been ascribed to post-transcriptional regulation including translational control or control of protein half life [42]. However, to measure the detailed correlation between the transcriptome and proteome of *A. fumigatus*, a parallel experiment comparing the entire proteome including all intracellular proteins to the transcriptome, needs to be performed.

The results described here may provide an intriguing explanation for why the *ΔprtT* mutant is normally virulent in a murine model of IPA, despite almost totally lacking secreted protease activity [11]. In the lungs, lacking the ability to derive nutrients from its protein-rich surroundings, the *ΔprtT* mutant may activate compensatory pathways leading, as we show in vitro, to the production of novel secondary metabolites and increased secretion of proteins not normally produced by the WT strain. These factors could raise its virulence to WT levels, despite its inability to produce secreted proteases. In fact, similar compensatory mechanisms may explain puzzling results described previously, where *A. fumigatus* mutants lacking what appear to be crucial virulence factors such as those affecting resistance to oxygen radicals [43], [44] or key signaling pathways significantly affecting fungal morphology [45], [46] were not decreased in virulence.

In summary, further characterization of the PrtT transcription factor showed that in addition to being primarily involved in activating the expression of secreted proteases, it is also involved in activating most of the genes for iron uptake and ergosterol biosynthesis, as well as inhibiting four secondary metabolite clusters and affecting expression of secreted polysaccharide-degrading enzymes. This study underscores the complex regulatory role and multiple redundant functions of a key fungal transcription factor.

## Supporting Information

Supporting Information S1 **Genes up or down-regulated in the** ***ΔprtT*** **mutant strain relative to the WT.**
(XLSX)

Supporting Information S2 **Supplemental Tables A-E.** Table A. Oligonucleotides used in this study. Table B. Selected enriched downregulated genes classes in the *ΔprtT* mutant vs. WT, genes that appear in more than one class are shown in the most specific class. Table C. Selected Enriched upregulated genes classes in the *ΔprtT* mutant vs.WT. Table D. Significantly enriched physical gene clusters upregulated or downregulated in the *ΔprtT* mutant vs. WT. Table E. Identified proteins and peptides by 2D-DIGE MS/MS.
(DOC)
