# Supplementary material for: Transcriptional and Proteomic Analysis of the Aspergillus fumigatus ΔprtT Protease-Deficient Mutant
Source: PLoS One. 2012 Apr 13;7(4):e33604. doi: 10.1371/journal.pone.0033604 (PMC3326020; doi:10.1371/journal.pone.0033604)
Supplement: Supporting Information S2 — Supplemental Tables A-E. Table A. Oligonucleotides used in this study. Table B. Selected enriched downregulated genes classes in the ΔprtT mutant vs. WT, genes that appear in more than one class are shown in the most specific class. Table C. Selected Enriched upregulated genes classes in the ΔprtT mutant vs.WT. Table D. Significantly enriched physical gene clusters upregulated or downregulated in the ΔprtT mutant vs. WT. Table E. Identified proteins and peptides by 2D-DIGE MS/MS. (DOC) [file pone.0033604.s002.doc]

Supplementary Tables

**Table A.** Oligonucleotides used in this study**.**

| **Oligonucleotide** | **Sequence (5'→3')** |
| --- | --- |
| AFUA_4G11800 (Alp1) 5' | TGACATTGTGAGCAAGGGTCGT |
| AFUA_4G11800 (Alp1) 3' | TCGAAAGCGTTCTCAACAGCGT |
| AFUA_2G07680 (SidA) 5' | TGGAGTTCGAGGACTACATG |
| AFUA_2G07680 (SidA) 3' | TCCCAACTCACGAACAACGCT |
| AFUA_3G03640 (MirB) 5' | ACCTACGCCAAGCCGAGAAA |
| AFUA_3G03640 (MirB) 3' | AGAACTCCGGGCAGATCAAAT |
| AFUA_4G06890 (Erg11) 5' | TGGACTATCTGCGCGATTCA |
| AFUA_4G06890 (Erg11) 3' | TGGAACGAACTTCCTGGCCTT |
| AFUA_2G 00320 (Erg3) 5' | TTCATCACGATCTGGGCTTTC |
| AFUA_2G 00320 (Erg3) 3' | GTTGCATGGCACGTTTGTCGA |
| AFUA_5G11260 (SreA) 5' | AACACACCACGGATCGTCAT |
| AFUA_5G11260 (SreA) 3' | TCAGCTGGAGGAGCAGTTCT |
| AFUA_5G03920 (HapX) 5' | TCAAGGAACAGATCACCAACCT |
| AFUA_5G03920 (HapX) 3' | ACCGATACCTCCTTCTCCA |
| AFUA_ 2G01260 (SrbA) 5' | TGCTGAGCTAAGAGACAGTGT |
| AFUA_ 2G01260 (SrbA) 3' | AGGATGGACGCTTTGTTGAGT |
| AFUA_2G12330 (AcuM) 5' | TCAGACTTGCTCCTTCGTGT |
| AFUA_2G12330 (AcuM) 3' | TGCCGTTCTCCGTCATCAA |
| AFUA_1G10910 (β-tubulin) 5' | TGGACGTTACCTCACCTGCT |
| AFUA_1G10910 (β-tubulin) 3' | TAGCTCTGGTTCTTGGACTGGA |

**Table B**. Selected enriched downregulated genes classes in the *ΔprtT* mutant vs. WT. Genes that appear in more than one class are shown in the most specific class.

| **Enriched class** | **Gene ID** | **Gene description** | **Fold- change** |
| --- | --- | --- | --- |
| **Secreted proteases *P*=0.002** | AFUA_4G11800 | alkaline serine protease Alp1 | 14.3 |
|  | AFUA_6G00310 | serine carboxypeptidase (CpdS), putative | 3.6 |
|  | AFUA_7G04930 | alkaline serine protease (PR1), putative | 2.5 |
|  | AFUA_2G17330 | serine peptidase, family S28, putative | 2.2 |
|  | AFUA_8G07080 | elastinolytic metalloproteinase Mep | 2.1 |
|  | AFUA_2G09030 | secreted dipeptidyl peptidase (DPPV) | 1.9 |
| **Iron uptake *P*=1E-15** | AFUA_3G03440* | MFS family siderophore transporter, putative | 39.8 |
|  | AFUA_7G06060 | siderochrome-iron transporter (Sit1), putative | 30.5 |
|  | AFUA_3G03640 | siderochrome-iron transporter (MirB), putative | 28.8 |
|  | AFUA_7G04730 | siderochrome-iron transporter, putative | 23.9 |
|  | AFUA_2G07680 | L-ornithine N5-oxygenase SidA | 23.1 |
|  | AFUA_5G03800 | high-affinity iron permease CaFTR2 | 16.6 |
|  | AFUA_3G03420* | SidD | 14.9 |
|  | AFUA_3G03400* | siderophore biosynthesis acetylase AceI, (SidF) | 9.2 |
|  | AFUA_1G17270 | ferric-chelate reductase (Fre2), putative | 8.3 |
|  | AFUA_3G03390* | siderophore biosynthesis lipase/esterase, putative | 5.0 |
|  | AFUA_2G05730 | siderochrome-iron transporter (36), putative | 3.5 |
|  | AFUA_3G03650 | acetyltransferase, GNAT family, putative Hass: SidG | 3.3 |
|  | AFUA_2G07670 | palmitoyltransferase (SidR), putative | 3.1 |
|  | AFUA_1G17200 | nonribosomal peptide synthase, putative (SidC) | 2.2 |
|  | AFUA_3G03450* | cytochrome P450 pisatin demethylase, putative | 2.1 |
| **Steroid and fatty acid biosynthesis *P*= 0.001** | AFUA_2G00320 | sterol delta 5,6-desaturase, putative | 6.5 |
|  | AFUA_4G06890^1^ | 14-alpha sterol demethylase Cyp51A | 4.2 |
|  | AFUA_1G03150^1^ | c-14 sterol reductase ERG4/ERG24 Family | 4.2 |
|  | AFUA_5G07780^1^ | squalene monooxygenase Erg1 | 3.7 |
|  | AFUA_8G02440^1^ | C-4 methyl sterol oxidase, putative ERG25 homolog | 3.6 |
|  | AFUA_6G05140^1^ | sterol delta 5,6-desaturase ERG3 | 2.9 |
|  | AFUA_1G15820 | fatty acid hydroxylase, putative | 2.4 |
|  | AFUA_7G03740^1^ | 14-alpha sterol demethylase Cyp51B | 2.3 |
|  | AFUA_1G16850 | sur2 protein sphinganine hydroxylase | 2.1 |
|  | AFUA_4G06940 | Sphingolipid desaturase (Des-1) | 2.1 |
|  | AFUA_6G00740 | hypothetical protein | 2 |
| **Cellular ketone metabolic process *P*=0.001** | AFUA_2G04200 | 4-hydroxyphenylpyruvate dioxygenase, putative | 4.6 |
|  | AFUA_2G04220^2^ | homogentisate 1,2-dioxygenase (HmgA), putative | 4.2 |
|  | AFUA_6G07720 | phosphoenolpyruvate carboxykinase (ATP) (AcuF) | 4 |
|  | AFUA_2G08280 | NADP-dependent malic enzyme (MaeA) | 3.5 |
|  | AFUA_6G08760^2^ | proline oxidase (PrnD) | 3.5 |
|  | AFUA_4G06620^2^ | Glutamate/Leucine/Phenylalanine/Valine dehydrogenase, putative | 3.3 |
|  | AFUA_2G04230^2^ | fumarylacetoacetate hydrolase (FahA) | 3.3 |
|  | AFUA_5G04250 | homocysteine synthase, putative | 3.1 |
|  | AFUA_6G08750^2^ | delta-1-pyrroline-5-carboxylate dehydrogenase (PrnC) | 3 |
|  | AFUA_2G01890 | Ubiquinone biosynthesis protein Coq7 | 2.9 |
|  | AFUA_2G05060 | alternative oxidase | 2.7 |
|  | AFUA_4G10410^2^ | aspartate aminotransferase, putative | 2.1 |
|  | AFUA_6G02470^2^ | fumarate hydratase, putative | 2 |
|  | AFUA_3G11430^2^ | arginase | 2 |
| **Oxidoreductase activity *P* = 0.008** | AFUA_1G17180 | pyridine nucleotide-disulphide oxidoreductase, putative | 7.7 |
|  | AFUA_5G03930 | alcohol dehydrogenase, putative | 3.8 |
|  | AFUA_6G13790 | monooxygenase | 3.6 |
|  | AFUA_7G02010 | hypothetical protein | 3.6 |
|  | AFUA_1G07480 | coproporphyrinogen III oxidase, putative | 3.3 |
|  | AFUA_7G06780 | hypothetical protein | 3.2 |
|  | AFUA_4G08710 | short chain dehydrogenase, putative | 3.2 |
|  | AFUA_8G05140 | oxidoreductin | 3 |
|  | AFUA_3G01900 | conserved hypothetical protein | 2.7 |
|  | AFUA_3G01580* | GMC oxidoreductase, putative | 2.6 |
|  | AFUA_5G10650 | pyridoxamine phosphate oxidase, putative | 2.4 |
|  | AFUA_7G05070 | FAD dependent oxidoreductase, putative | 2.4 |
|  | AFUA_3G03280* | FAD binding monooxygenase, putative | 2.1 |
|  | AFUA_1G17170 | alpha-ketoglutarate-dependent taurine dioxygenase | 2.1 |
|  | AFUA_1G14340 | metalloreductase, putative | 2.1 |
|  | AFUA_6G08440 | protoporphyrinogen oxidase, putative | 2.1 |
|  | AFUA_6G04920 | NAD-dependent formate dehydrogenase | 2 |
| **Cytochrome C oxidoreductase activity *P* = 0.008** | AFUA_3G06190 | Cytochrome c oxidase subunit VIa family | 2.6 |
|  | AFUA_2G03010 | cytochrome c subunit Vb, putative | 2.5 |
|  | AFUA_3G14440 | cytochrome c oxidase family protein | 2.6 |
|  | AFUA_5G10560 | cytochrome c oxidase subunit V | 2.2 |

^1^Involved in ergosterol biosynthesis.

^2^Involved in amino acid catabolism.

* Localized to a gene cluster.

Table C: Selected Enriched upregulated genes classes in the *ΔprtT* mutant vs. WT.

| **Enriched class** | **Gene ID** | **Gene description** | **Fold- change** |
| --- | --- | --- | --- |
| **Transporter activity *P* = 0.008** | AFUA_8G00540* | hybrid polyketide synthase/nonribosomal peptide synthase, pseurotin biosynthesis | 43.1 |
|  | AFUA_8G00370* | polyketide synthase, putative | 27.7 |
|  | AFUA_8G00940 | MFS drug transporter, aflatoxin exporter | 21.0 |
|  | AFUA_8G00800 | amino acid transporter, putative | 9.9 |
|  | AFUA_2G09860 | purine-cytosine permease | 8.1 |
|  | AFUA_6G11840 | sodium:bile acid symporter involved in azole resistance | 5.4 |
|  | AFUA_4G01230 | amino acid transporter, putative | 5.2 |
|  | AFUA_6G03060 | MFS monosaccharide transporter | 5.0 |
|  | AFUA_1G12240 | MFS peptide transporter, putaitve | 4.6 |
|  | AFUA_5G11020 | ammonium transporter | 4.3 |
|  | AFUA_3G12920* | nonribosomal peptide synthase NRPS5/pesF | 4.2 |
|  | AFUA_5G13170 | MATE efflux family protein subfamily, putative | 3.6 |
|  | AFUA_6G07260 | purine-cytosine permease, putative | 3.3 |
|  | AFUA_2G10560 | amino acid permease | 3.1 |
|  | AFUA_3G11790 | galactose-proton symport, putative | 3.1 |
|  | AFUA_5G06230 | gaba-specific permease | 2.8 |
|  | AFUA_1G12850 | CrnA, nitrate transporter (nitrate permease) | 2.8 |
|  | AFUA_1G14330 | abcC, ABC transporter, putative | 2.7 |
|  | AFUA_3G12220 | ABC transporter, putative | 2.7 |
|  | AFUA_5G02780 | mitochondrial nicotinamide nucleotide transhydrogenase subunit, putative | 2.7 |
|  | AFUA_4G00800 | MFS monosaccharide transporter, putative | 2.7 |
|  | AFUA_1G17470 | high affinity nitrate transporter NrtB | 2.2 |
|  | AFUA_6G13190 | H+/nucleoside cotransporter | 2.0 |
|  | AFUA_6G03200 | solute symporter family transporter | 1.8 |
|  | AFUA_6G07750 | MFS phospholipid transporter (Git1), putative | 1.8 |
|  | AFUA_1G04870 | urea transporter (Dur3), putative | 1.5 |
| **Oxidoreductase activity *P* = 0.04** | AFUA_8G00480* | phytanoyl-CoA dioxygenase family protein | 41.9 |
|  | AFUA_8G00560* | cytochrome P450, similar to SP:P79084: O-methylsterigmatocystin oxidoreductase (*Aspergillus flavus*) | 38.1 |
|  | AFUA_8G00440* | steroid monooxygenase, putative | 15.8 |
|  | AFUA_6G11850 | hypothetical protein | 11.4 |
|  | AFUA_4G14780* | cyp5081A1 cytochrome P450 monooxygenase, putative | 10.3 |
|  | AFUA_5G08900 | D-arabinitol dehydrogenase ArbD, putative | 9.5 |
|  | AFUA_4G14800* | sdr1 short chain dehydrogenase, putative | 9.5 |
|  | AFUA_1G04150 | tartrate dehydrogenase | 8.9 |
|  | AFUA_3G12960* | cytochrome P450 monooxigenase (GliC ortholog), putative | 8.6 |
|  | AFUA_8G00510* | O-methylsterigmatocystin oxidoreductase, putative | 7.0 |
|  | AFUA_6G03440* | fructosyl amine oxidase | 6.8 |
|  | AFUA_2G14480 | oxidoreductase, FAD-binding, putative | 6.6 |
|  | AFUA_3G00800 | oxidoreductase, 2OG-Fe(II) oxygenase family, putative | 6.5 |
|  | AFUA_6G12070* | FAD binding domain protein | 6.0 |
|  | AFUA_4G14790* | cyp5081B1 cytochrome P450 monooxygenase, putative | 5.7 |
|  | AFUA_2G17540* | pigment biosynthesis oxidase Abr1/brown 1 | 5.7 |
|  | AFUA_6G13970* | FAD-dependent monooxygenase (PaxM), putative | 5.2 |
|  | AFUA_7G01000 | aldehyde dehydrogenase, putative | 4.8 |
|  | AFUA_6G11430 | aldA, aldehyde dehydrogenase, putative | 4.4 |
|  | AFUA_4G14830* | cytochrome P450 monooxygenase, putative | 4.0 |
|  | AFUA_4G14850* | extracellular 3-ketosteroid 1-dehydrogenase, putative | 3.6 |
|  | AFUA_6G13940* | P450-monooxygenase, putative | 3.5 |
|  | AFUA_4G14810* | cyp5081D1 cytochrome P450 monooxygenase, putative | 3.1 |
|  | AFUA_5G12470 | oxidoreductase, 2OG-Fe(II) oxygenase family | 2.9 |
|  | AFUA_5G02620 | cytochrome P450, putative | 2.8 |
|  | AFUA_5G02780 | mitochondrial nicotinamide nucleotide transhydrogenase subunit, putative | 2.7 |
|  | AFUA_8G00280* | short-chain dehydrogenase, putative | 2.6 |
|  | AFUA_4G08600 | aldehyde dehydrogenase, putative | 2.6 |
|  | AFUA_2G14470 | oxidoreductase, FAD-binding, putative | 2.4 |
|  | AFUA_4G11730 | glycerol dehydrogenase (GldB), putative | 2.4 |
|  | AFUA_6G08920 | assimilatory sulfite reductase | 2.4 |
|  | AFUA_3G11930 | NAD binding Rossmann fold oxidoreductase, putative | 2.4 |
|  | AFUA_8G05530 | soluble fumarate reductase (Osm1), putative | 2.3 |
|  | AFUA_8G06440 | fructosyl amino acid oxidase, putative | 2.2 |
|  | AFUA_4G11220 | xanthine dehydrogenase | 2.2 |
|  | AFUA_1G00500 | FMN dependent dehydrogenase, putative | 2.0 |
|  | AFUA_5G07000 | NAD binding Rossmann fold oxidoreductase, putative | 2.0 |
|  | AFUA_6G09140 | oxidoreductase, short-chain dehydrogenase/reductase family | 2.0 |
|  | AFUA_7G06630 | cytochrome P450 monooxygenase, putative | 2.0 |
|  | AFUA_5G07360 | peroxisomal copper amine oxidase | 1.8 |
|  | AFUA_4G09110 | cytochrome c peroxidase, putative | 1.8 |
|  | AFUA_3G08470 | glucose-6-phosphate 1-dehydrogenase | 1.5 |

* Localized to a gene cluster

Table D. Significantly enriched physical clusters differentially up or downregulated in the *ΔprtT* mutant vs. WT.

| Group | Putative function | P-value | # genes | Genes (fold change) |
| --- | --- | --- | --- | --- |
| Downregulated | Siderophore biosynthetic cluster | < E-7 | 9 | AFUA_3G03440(-39.8) |
|  |  |  |  | AFUA_3G03420(-14.9) |
|  |  |  |  | AFUA_3G03410(-14.9) |
|  |  |  |  | AFUA_3G03430(-11.1) |
|  |  |  |  | AFUA_3G03400(-9.2) |
|  |  |  |  | AFUA_3G03390(-5.0) |
|  |  |  |  | AFUA_3G03580(-2.7) |
|  |  |  |  | AFUA_3G03570(-2.3) |
|  |  |  |  | AFUA_3G03450(-2.1) |
|  |  |  |  |  |
| Upregulated | ETP unknown Toxin Biosynthesis Cluster | < E-6 | 7 | AFUA_3G12900(32.3) |
|  |  |  |  | AFUA_3G12890(10.1) |
|  |  |  |  | AFUA_3G12960(6.3) |
|  |  |  |  | AFUA_3G12930(3.3) |
|  |  |  |  | AFUA_3G12920(3.3) |
|  |  |  |  | AFUA_3G12910(3.0) |
|  |  |  |  | AFUA_3G13010(2.3) |
| Upregulated | Unknown | < E-5 | 10 | AFUA_4G14770(9.8) |
|  |  |  |  | AFUA_4G14780(8.1) |
|  |  |  |  | AFUA_4G14800(6.9) |
|  |  |  |  | AFUA_4G14530(5.3) |
|  |  |  |  | AFUA_4G14790(5.0) |
|  |  |  |  | AFUA_4G14820(4.3) |
|  |  |  |  | AFUA_4G14840(4.2) |
|  |  |  |  | AFUA_4G14830(3.5) |
|  |  |  |  | AFUA_4G14850(3.4) |
|  |  |  |  | AFUA_4G14810(3.0) |
| Upregulated | Unknown | < E-3 | 4 | AFUA_6G13980(4.3) |
|  |  |  |  | AFUA_6G13940(3.9) |
|  |  |  |  | AFUA_6G13920(3.8) |
|  |  |  |  | AFUA_6G13970(3.6) |
| Upregulated | Pseurotin biosynthetic cluster | < E-15 | 23 | AFUA_8G00480(30.1) |
|  |  |  |  | AFUA_8G00540(29.2) |
|  |  |  |  | AFUA_8G00560(29.0) |
|  |  |  |  | AFUA_8G00550(28.2) |
|  |  |  |  | AFUA_8G00390(26.6) |
|  |  |  |  | AFUA_8G00580(25.0) |
|  |  |  |  | AFUA_8G00430(22.1) |
|  |  |  |  | AFUA_8G00370(21.4) |
|  |  |  |  | AFUA_8G00400(20.3) |
|  |  |  |  | AFUA_8G00520(20.2) |
|  |  |  |  | AFUA_8G00570(13.3) |
|  |  |  |  | AFUA_8G00440(12.5) |
|  |  |  |  | AFUA_8G00490(10.0) |
|  |  |  |  | AFUA_8G00530(9.6) |
|  |  |  |  | AFUA_8G00500(8.5) |
|  |  |  |  | AFUA_8G00420(7.8) |
|  |  |  |  | AFUA_8G00510(5.1) |
|  |  |  |  | AFUA_8G00470(3.4) |
|  |  |  |  | AFUA_8G00380(3.4) |
|  |  |  |  | AFUA_8G00360(2.5) |
|  |  |  |  | AFUA_8G00280(2.2) |
|  |  |  |  | AFUA_8G00460(2.2) |
|  |  |  |  | AFUA_8G00410(2.1) |

**Table E**. Identified proteins and peptides by 2D-DIGE MS/MS.

| **Master (DeCyder)** | **Identified Protein by MS/MS** | **UniProt Accession Nr.** | **Cov (%)** | **Number of Peptides** | **MW theoretical (kDa)/p*I*** | **Identified peptides** |
| --- | --- | --- | --- | --- | --- | --- |
| 32 | Secreted dipeptidyl peptidase DppV | P0C959 | 16 | 11 | 79.6/5.59 | 41 - 55 K.VAVFSTSQYSFETHK.R |
|  |  |  |  |  |  | 41 - 56 K.VAVFSTSQYSFETHKR.T |
|  |  |  |  |  |  | 57 - 66 R.TSWWSLLDLK.T |
|  |  |  |  |  |  | 120 - 131 K.AASLPASFSGLK.T |
|  |  |  |  |  |  | 169 - 176 R.IYDSIYVR.H |
|  |  |  |  |  |  | 302 - 308 K.LAYFQMR.D |
|  |  |  |  |  |  | 329 - 339 K.TIPSVAGDWDR.S |
|  |  |  |  |  |  | 352 - 362 K.TLIVGSEDLGR.T |
|  |  |  |  |  |  | 594 - 604 K.VSTEELWFMQR.E |
|  |  |  |  |  |  | 628 - 645 R.ILQFATPMLVIHSDKDYR.L |
|  |  |  |  |  |  | 646 - 661 R.LPVAEGLSLFNVLQER.G |
| 35 | Secreted dipeptidyl peptidase DppV | P0C959 | 22 | 15 | 79.6/5.59 | 41 - 55 K.VAVFSTSQYSFETHK.R |
|  |  |  |  |  |  | 41 - 56 K.VAVFSTSQYSFETHKR.T |
|  |  |  |  |  |  | 120 - 131 K.AASLPASFSGLK.T |
|  |  |  |  |  |  | 169 - 176 R.IYDSIYVR.H |
|  |  |  |  |  |  | 218 – 239 K.NAESPYPPFGGASDYDLSPDGK.W |
|  |  |  |  |  |  | 302 - 308 K.LAYFQMR.D |
|  |  |  |  |  |  | 309 - 317 R.DETYESDRR.V |
|  |  |  |  |  |  | 318 - 327 R.VLYVYSLGSK.K |
|  |  |  |  |  |  | 352 - 362 K.TLIVGSEDLGR.T |
|  |  |  |  |  |  | 365 - 373 R.LFSLPANAK.D |
|  |  |  |  |  |  | 424 - 435 K.IASANEIDPELK.G |
|  |  |  |  |  |  | 580 - 593 K.ALVSHDGTFVADAK.V |
|  |  |  |  |  |  | 628 - 642 R.ILQFATPMLVIHSDK.D |
|  |  |  |  |  |  | 628 - 645 R.ILQFATPMLVIHSDKDYR.L |
|  |  |  |  |  |  | 646 - 661 R.LPVAEGLSLFNVLQER.G |
| 30 | Secreted dipeptidyl peptidase DppV | P0C959 | 6 | 4 | 79.6/5.59 | 169 - 176 R.IYDSIYVR.H |
|  |  |  |  |  |  | 302 - 308 K.LAYFQMR.D |
|  |  |  |  |  |  | 628 - 645 R.ILQFATPMLVIHSDKDYR.L |
|  |  |  |  |  |  | 646 - 661 R.LPVAEGLSLFNVLQER.G |
| 21 | Secreted dipeptidyl peptidase | P0C959 | 22 | 13 | 79.6/5.59 | 41 - 55 K.VAVFSTSQYSFETHK.R |
|  |  |  |  |  |  | 41 - 56 K.VAVFSTSQYSFETHKR.T |
|  |  |  |  |  |  | 120 - 131 K.AASLPASFSGLK.A |
|  |  |  |  |  |  | 169 - 176 R.IYDSIYVR.H |
|  |  |  |  |  |  | 218 - 239 K.NAESPYPPFGGASDYDLSPDGK.W |
|  |  |  |  |  |  | 302 - 308 K.LAYFQMR.D |
|  |  |  |  |  |  | 309 - 327 R.DETYESDRALLYVYSLGSK.K |
|  |  |  |  |  |  | 352 - 362 K.TLIVGSEDLGR.T |
|  |  |  |  |  |  | 365 - 373 R.LFSLPANAK.D |
|  |  |  |  |  |  | 424 - 435 K.IASANEIDPELK.G |
|  |  |  |  |  |  | 580 - 593 K.ALVSHDGTFVADAK.V |
|  |  |  |  |  |  | 628 - 642 R.ILQFATPMLVIHSDK.D |
|  |  |  |  |  |  | 646 - 661 R.LPVAEGLSLFNVLQER.G |
| 94 | Pheromone processing carboxypeptidase (Sxa2) | Q5VJG7 | 8 | 4 | 59.7/4.77 | 79 - 93 K.FDIGEMYSGSVPIDK.G |
|  |  |  |  |  |  | 194 - 202 K.NFQEIFGIK.N |
|  |  |  |  |  |  | 206 - 216 K.IYVTGESYAGR.Y |
|  |  |  |  |  |  | 295 - 307 K.YLVFPPSGVQPPK.M |
|  | Tripeptidyl-peptidase (TppA) | Q70J59 | 9 | 4 | 65.7/5.3 | 104 - 122 R.GWLESAGISDIEEDADWIK.F |
|  |  |  |  |  |  | 220 - 228 K.DLYNIGDYK.A |
|  |  |  |  |  |  | 237 - 248 K.VAFASFLEEYAR.Y |
|  |  |  |  |  |  | 477 - 493 R.AFPDVAAQAENYAVFDK.G |
| 87 | Pheromone processing carboxypeptidase (Sxa2) | Q5VJG7 | 17 | 8 | 59.7/4.77 | 65 - 72 K.DTKPYLVK.S |
|  |  |  |  |  |  | 73 – 93 K.SLPDVKFDIGEMYSGSVPIDK.G |
|  |  |  |  |  |  | 79 - 93 K.FDIGEMYSGSVPIDK.G |
|  |  |  |  |  |  | 194 – 202 K.NFQEIFGIK.N |
|  |  |  |  |  |  | 206 - 216 K.IYVTGESYAGR.Y |
|  |  |  |  |  |  | 217 – 237 R.YVPYISAAMLDQNDTEYYDLK.G |
|  |  |  |  |  |  | 282 - 294 R.LHQTCGYQDFIDK.Y |
|  |  |  |  |  |  | 295 – 307 K.YLVFPPSGVQPPK.M |
| 110 | Serine peptidase | Q4WIN2 | 5 | 2 | 58.5/4.86 | 103 - 117 R.FAEEIGGAVILLEHR.Y |
|  |  |  |  |  |  | 230 - 245 R.VVEYIDHVYESGDIER.Q |
| 168 | Mannosidase I | Q6PWQ1 | 24 | 10 | 55.4/5.14 | 50 - 68 K.YAFPHDELLPVSNSYGDSR.N |
|  |  |  |  |  |  | 106 - 118 K.TSDMVSLFETTIR.Y |
|  |  |  |  |  |  | 119 - 131 R.YLSGMLSGYDLLK.G |
|  |  |  |  |  |  | 201 - 212 R.LSDLTGDQEYAK.L |
|  |  |  |  |  |  | 275 - 280 K.RFATYK.D |
|  |  |  |  |  |  | 375 - 386 K.SVPADQKELYER.A |
|  |  |  |  |  |  | 432 - 453 K.YCRTESGFAGLTNVNAVNGGGR.Y |
|  |  |  |  |  |  | 435 - 453 R.TESGFAGLTNVNAVNGGGR.Y |
|  |  |  |  |  |  | 454 - 467 R.YDNQESFLFAEVMK.Y |
|  |  |  |  |  |  | 489 – 499 K.FVYNTEAHPVR.I |
|  | FAD-dependent oxygenase | Q4WFW0 | 14 | 6 | 54.9/6.52 | 54 - 68 K.IYFPGSTEFEQASTR.W |
|  |  |  |  |  |  | 259 - 272 K.VEAVYQAANEHLLK.N |
|  |  |  |  |  |  | 360 - 365 R.FPIYLR.E |
|  |  |  |  |  |  | 374 - 384 K.TAYDIFAAATR.G |
|  |  |  |  |  |  | 411 - 417 K.SSAFAFR.N |
|  |  |  |  |  |  | 418 - 436 R.NENLLAAPLVTYAPAGPER.D |
| 163 | FAD-dependent oxygenase | Q4WFW0 | 14 | 5 | 54.9/6.52 | 54 - 68 K.IYFPGSTEFEQASTR.W |
|  |  |  |  |  |  | 259 - 272 K.VEAVYQAANEHLLK.N |
|  |  |  |  |  |  | 374 - 384 K.TAYDIFAAATR.G |
|  |  |  |  |  |  | 406 - 417 K.AINSKSSAFAFR.N |
|  |  |  |  |  |  | 418 - 436 R.NENLLAAPLVTYAPAGPER.D |
| 164 | FAD-dependent oxygenase | Q4WFW0 | 15 | 6 | 54.9/6.52 | 54 - 68 K.IYFPGSTEFEQASTR.W |
|  |  |  |  |  |  | 259 - 272 K.VEAVYQAANEHLLK.N |
|  |  |  |  |  |  | 366 - 373 R.EYNVPAQK.T |
|  |  |  |  |  |  | 374 - 384 K.TAYDIFAAATR.G |
|  |  |  |  |  |  | 406 - 417 K.AINSKSSAFAFR.N |
|  |  |  |  |  |  | 418 - 436 R.NENLLAAPLVTYAPAGPER.D |
|  | Mannosidase I | Q6PWQ1 | 26 | 10 | 55.4/5.14 | 38 - 49 K.EAFSHAWDGYMK.Y |
|  |  |  |  |  |  | 50 - 68 K.YAFPHDELLPVSNSYGDSR.N |
|  |  |  |  |  |  | 106 - 118 K.TSDMVSLFETTIR.Y |
|  |  |  |  |  |  | 119 - 131 R.YLSGMLSGYDLLK.G |
|  |  |  |  |  |  | 201 - 212 R.LSDLTGDQEYAK.L |
|  |  |  |  |  |  | 432 – 453 K.YCRTESGFAGLTNVNAVNGGGR.Y |
|  |  |  |  |  |  | 435 - 453 R.TESGFAGLTNVNAVNGGGR.Y |
|  |  |  |  |  |  | 454 - 467 R.YDNQESFLFAEVMK.Y |
|  |  |  |  |  |  | 468 - 483 K.YAYLTHAPEDEWQVQR.G |
|  |  |  |  |  |  | 489 - 499 K.FVYNTEAHPVR.I |
| 148 | FAD-dependent oxygenase | Q4WFW0 | 18 | 7 | 54.9/6.52 | 54 - 68 K.IYFPGSTEFEQASTR.W |
|  |  |  |  |  |  | 100 - 118 K.NLPFLAYNGVHGAITTLGR.M |
|  |  |  |  |  |  | 259 - 272 K.VEAVYQAANEHLLK.N |
|  |  |  |  |  |  | 366 – 373 R.EYNVPAQK.T |
|  |  |  |  |  |  | 374 - 384 K.TAYDIFAAATR.G |
|  |  |  |  |  |  | 418 - 436 R.NENLLAAPLVTYAPAGPER.D |
|  |  |  |  |  |  | 500 – 507 K.FSFYGPIA.- |
| 187 | FAD-dependent oxygenase | Q4WFW0 | 21 | 8 | 54.9/6.52 | 54 - 68 K.IYFPGSTEFEQASTR.W |
|  |  |  |  |  |  | 100 - 118 K.NLPFLAYNGVHGAITTLGR.M |
|  |  |  |  |  |  | 239 - 258 K.IYDIQHADWAIDTLIFSGDK.V |
|  |  |  |  |  |  | 259 - 272 K.VEAVYQAANEHLLK.N |
|  |  |  |  |  |  | 374 - 384 K.TAYDIFAAATR.G |
|  |  |  |  |  |  | 411 - 436 K.SSAFAFRNENLLAAPLVTYAPAGPER.D |
|  |  |  |  |  |  | 418 - 436 R.NENLLAAPLVTYAPAGPER.D |
|  |  |  |  |  |  | 418 - 441 R.NENLLAAPLVTYAPAGPERDNDAK.Q |
| 188 | FAD-dependent oxygenase | Q4WFW0 | 23 | 9 | 54.9/6.52 | 54 - 68 K.IYFPGSTEFEQASTR.W |
|  |  |  |  |  |  | 100 - 118 K.NLPFLAYNGVHGAITTLGR.M |
|  |  |  |  |  |  | 119 - 137 R.MDHGIEIYLNQLSGVEISK.D |
|  |  |  |  |  |  | 259 – 272 K.VEAVYQAANEHLLK.N |
|  |  |  |  |  |  | 366 - 373 R.EYNVPAQK.T |
|  |  |  |  |  |  | 374 - 384 K.TAYDIFAAATR.G |
|  |  |  |  |  |  | 418 - 436 R.NENLLAAPLVTYAPAGPER.D |
|  |  |  |  |  |  | 418 - 441 R.NENLLAAPLVTYAPAGPERDNDAK.Q |
|  |  |  |  |  |  | 500 - 507 K.FSFYGPIA.- |
| 211 | Tripeptidyl-peptidase (TppA) | Q70J59 | 12 | 7 | 65.7/5.30 | 104 - 122 R.GWLESAGISDIEEDADWIK.F |
|  |  |  |  |  |  | 220 - 228 K.DLYNIGDYK.A |
|  |  |  |  |  |  | 237 – 248 K.VAFASFLEEYAR.Y |
|  |  |  |  |  |  | 370 – 381 R.SVCNLYAQLGSR.G |
|  |  |  |  |  |  | 370 - 381 R.SVCNLYAQLGSR.G |
|  |  |  |  |  |  | 467 - 474 R.YKGLYNPK.G |
|  |  |  |  |  |  | 477 - 493 R.AFPDVAAQAENYAVFDK.G |
| 236 | GPI-anchored cell wall beta-1,3-endoglucanase EglC | Q4WG16 | 20 | 7 | 44.6/4.90 | 36 - 48 K.SQADFQAEFSTAK.N |
|  |  |  |  |  |  | 49 - 60 K.NLVGTSGFTSAR.L |
|  |  |  |  |  |  | 107 - 118 K.AAIAQYGDDLAK.L |
|  |  |  |  |  |  | 119 - 132 K.LVVGISVGSEDLYR.N |
|  |  |  |  |  |  | 140 – 159 K.ANAGIGTNPDEIVSYINEVR.S |
|  |  |  |  |  |  | 217 - 225 K.ALFDESVAK.T |
|  |  |  |  |  |  | 261 - 273 K.TYWDEVGCPLFGK.T |
|  | 1,3-beta-glucanosyltransferase Bgt1 | Q4WSV9 | 32 | 8 | 33/5.02 | 39 - 51 K.VQSDYETDFDTLK.E |
|  |  |  |  |  |  | 82 – 92 K.VVLGVWPDYDK.S |
|  |  |  |  |  |  | 93 - 102 K.SFTDDFNALK.E |
|  |  |  |  |  |  | 103 - 124 K.EAVPGNEEVIDAITVGSEVLYR.K |
|  |  |  |  |  |  | 126 - 135 K.SLTPQALLAR.I |
|  |  |  |  |  |  | 146 - 156 K.ITVGMVDSWNK.F |
|  |  |  |  |  |  | 274 - 294 K.TKGDNGEMQDETHWGAFTADR.K |
|  |  |  |  |  |  | 276 - 294 K.GDNGEMQDETHWGAFTADR.K |
| 237 | GPI-anchored cell wall beta-1,3-endoglucanase EglC | Q4WG16 | 13 | 5 | 44.6/4.90 | 36 - 48 K.SQADFQAEFSTAK.N |
|  |  |  |  |  |  | 49 - 60 K.NLVGTSGFTSAR.L |
|  |  |  |  |  |  | 107 - 118 K.AAIAQYGDDLAK.L |
|  |  |  |  |  |  | 119 - 132 K.LVVGISVGSEDLYR.N |
|  |  |  |  |  |  | 217 - 225 K.ALFDESVAK.T |
| 231 | GPI-anchored cell wall beta-1,3-endoglucanase EglC | Q4WG16 | 24 | 8 | 44.6/4.9 | 36 - 48 K.SQADFQAEFSTAK.N |
|  |  |  |  |  |  | 49 - 60 K.NLVGTSGFTSAR.L |
|  |  |  |  |  |  | 107 - 118 K.AAIAQYGDDLAK.L |
|  |  |  |  |  |  | 119 - 132 K.LVVGISVGSEDLYR.N |
|  |  |  |  |  |  | 140 - 159 K.ANAGIGTNPDEIVSYINEVR.S |
|  |  |  |  |  |  | 217 - 225 K.ALFDESVAK.T |
|  |  |  |  |  |  | 232 - 247 K.GKEVWITETGWPVSGK.T |
|  |  |  |  |  |  | 261 - 273 K.TYWDEVGCPLFGK.T |
| 318 | Alkaline serine protease Alp1 | P28296 | 32 | 7 | 42.1/6.32 | 128 – 139 K.GAPWGLGSISHK.G |
|  |  |  |  |  |  | 140 - 175 K.GQASTDYIYDTSAGAGTYAYVVDSGINVNHVEFESR.A |
|  |  |  |  |  |  | 176 - 204 R.ASLAYNAAGGSHVDSIGHGTHVAGTIGGK.T |
|  |  |  |  |  |  | 219 - 244 K.VFQGESSSTSIILDGFNWAVNDIVSK.G |
|  |  |  |  |  |  | 378 - 391 R.IKELATNGVVTNVK.G |
|  |  |  |  |  |  | 380 - 391 K.ELATNGVVTNVK.G |
|  |  |  |  |  |  | 392 - 403 K.GSPNKLAYNGNA.- |
| 334 | Alkaline serine protease Alp1 | P28296 | 16 | 5 | 42.1/6.32 | 128 - 139 K.GAPWGLGSISHK.G |
|  |  |  |  |  |  | 176 - 204 R.ASLAYNAAGGSHVDSIGHGTHVAGTIGGK.T |
|  |  |  |  |  |  | 378 - 391 R.IKELATNGVVTNVK.G |
|  |  |  |  |  |  | 380 - 391 K.ELATNGVVTNVK.G |
|  |  |  |  |  |  | 392 - 403 K.GSPNKLAYNGNA.- |
| 407 | Chitosanase | Q9Y760 | 36 | 5 | 21.5/5.76 | 20 – 37 K.SFSYCGDIPGAIFISSSK.G |
|  |  |  |  |  |  | 38 - 55 K.GYTNMDIDCDGANNSAGK.C |
|  |  |  |  |  |  | 56 – 69 K.CANDPSGQGETAFK.S |
|  |  |  |  |  |  | 74 - 98 K.KFGISDLDANIHPYVVFGNEDHSPK.F |
|  |  |  |  |  |  | 75 - 98 K.FGISDLDANIHPYVVFGNEDHSPK.F |
| 489 | Conserved hypothetical protein | Q4W9T1 | 16 | 1 | 15.9/5.71 | 45 - 68 K.HNDLYVYAYHTGAGFNDAVLTPDK.D |
